# Blood Pressure Variability in Stroke: Building a Framework, Conceptualizing Intervention Opportunities, and Identifying Practical Research Objectives

**DOI:** 10.1007/s12028-025-02389-9

**Published:** 2025-10-06

**Authors:** David Z. Rose, Alejandro A. Rabinstein, May Kim-Tenser, Sergio D. Bergese, Gabriel V. Fontaine, Charles Kircher, Adnan I. Qureshi

**Affiliations:** 1 University of South Florida Morsani College of Medicine, Tampa General Hospital, Tampa, FL USA; 2 Mayo Clinic, Rochester, MN USA; 3 Keck Medicine of USC, Los Angeles, CA USA; 4 Stony Brook University, Stony Brook, NY USA; 5 Intermountain Health, Salt Lake City, UT USA; 6 Washington University School of Medicine, St. Louis, MO USA; 7 Zeenat Qureshi Stroke Institute, Department of Neurology, University of Missouri, Columbia, MO USA

**Keywords:** ICH, Ischemic stroke, Neuromonitoring, Pharmacology, Artificial intelligence

## To the Editors of Neurocritical Care,

We, the members of the Blood Pressure Variability in Cerebrovascular Emergencies (B-PRECISE) consortia [1], thank our nephrology and critical care colleagues from Brazil [2] for their comments on our *Viewpoint* on blood pressure (BP) variability (BPV) in acute stroke [1]. We agree that BPV is “important and often underappreciated,” and we appreciate the compliment that our “efforts to establish a framework for BPV management are commendable.” Indeed, the point of our *Viewpoint* was trifold: we intended, first, to build this framework; second, to conceptualize intervention opportunities with data already published so far; and, third, to identify practical research objectives with the tools currently available at our disposal.

When we created our consensus statement, we held in-person discussions and multiple subsequent electronic communications in which we uniformly upheld the fixed and firm promise that nowhere would we (nor did we) state the word “recommendation” anywhere in the entire *Viewpoint* article; the only exception, of course, was when we specifically quoted or referenced American Heart Association/American Stroke Association (AHA/ASA) or European Stroke Organization “guideline recommendations” in the text. Repeatedly, we categorically affirmed that BPV thresholds require prospective validation before clinical application and that these concepts were intended to generate hypotheses for further study rather than serve as “recommendations” for immediate use [1]. This perspective is consistent with other reviews that diligently emphasize the value of prospective, disease-specific studies to determine whether BPV is a viable therapeutic target [3–5].

Although we agree our approach is “pragmatic,” we disagree that it “represents an oversimplification” of complex mathematical equations to calculate BPV that were “dismissed solely because of computational burden.” Paradoxically, their *Letter to the Editor* then states, “substantial implementation barriers remain” because “current electronic medical record systems cannot adequately capture real-time BP dynamics.” Here is the reality check: average real variability (ARV) and successive variation (SV) are not easily and readily calculated at the bedside; current technology even at the most advanced hospitals and with the most cutting-edge computational systems can neither routinely perform such calculations, nor integrate/act upon such data. This is why we emphasized starting simple, with systolic BP (SBP) range (maximum–minimum) for optimal feasibility across practice environments in future research instead of ARV or SV. Remarkably, artificial intelligence (AI) is advancing the fields of neurocardiology and neuroradiology already, so we expect advances in electronic monitoring and analytic tools to make more sophisticated indices, such as ARV and SV, practical, but, alas, that is not the case just yet [5, 6].

Regarding our framework distinguishing hyperacute SBP variability (SBPV1) and maintenance (SBPV2) phases, again, these were proposed as a conceptual scaffold to guide research, not as a prescriptive clinical protocol for today’s patients with stroke [1]. Developments in digital health using AI and other technology [5] can allow these concepts to be tested prospectively first, validated, and then implemented clinically—in that order, and not the other way around.

We agree that specific antihypertensive selection criteria need further evaluation and exploration, particularly short-acting intravenous agents. Large studies, such as INTERACT2 [7] and ATACH-2 [8], tested intensive SBP reduction strategies but were not designed to compare pharmacologic agents head-to-head or with *a priori* BPV primary outcomes. As such, no prospective evidence supports the preferential use of one agent over another to mitigate BPV. Theoretically, the shortest-acting intravenous antihypertensives could serve as optimal vehicles to titrate quickly at bedside, minimize BPV, improve outcomes, and free up nursing staff to perform other important tasks; however, our consensus explicitly acknowledged that this hypothesis requires investigation [3, 5]. Of interest is a subclass of intravenous short-acting nondihydropyridine calcium-channel blocker (CCB), called “ultra-short-acting” with a one-minute half-life. If such therapy could smoothly lower SBP in patients with stroke to within very narrow ranges—and maintain SBP within those tight ranges in the hyperacute and acute phases with fewer spikes in peaks and troughs than other agents—then they could potentially be a reproducible and generalizable option to reliably hit SBPV targets. In the open-label, phase 3b ACCELERATE clinical trial of the ultra-short-acting CCB clevidipine, patients with intracranial hemorrhage (ICH) and SBP > 160 mm Hg were given a prespecified, strict, 20–mm Hg target SBP goal range of 140–160 mm Hg on admission [9]. The median time-to-target SBP in ACCELERATE was 5.5 min, and 96.9% of study participants achieved this with clevidipine monotherapy (not requiring a rescue agent) [9]. The standard error around the measured SBP, essentially a marker of SBPV, narrowed to smaller and smaller standard error increments over time, and the target SBP range was maintained throughout the duration of the infusion [9]. Such predictable behavior aligns with the 2022 AHA/ASA guidelines, which recommend “careful titration to ensure continuous smooth and sustained control of BP, avoiding peaks and large variability in SBP.” These guidelines state that avoiding variability “can be beneficial for improving functional outcomes” (class of recommendation: 2a; level of evidence: B-nonrandomized) in patients with spontaneous ICH requiring acute BP lowering [10].

Another intravenous antihypertensive, nitroprusside, also reaches target SBP quickly, within about two minutes; however, its use in patients with stroke is discouraged because of its tendency to raise intracranial pressure [11]. The “intermediate-to-long acting” antihypertensives, such as fenoldopam and labetalol, and even the "short-acting" CCB nicardipine, have an onset of action within 5 to 15 min; however, their antihypertensive effects may last several hours—a serious drawback in case of overshoot resulting in persistent hypotension. Resuscitation with copious intravenous fluids or pressor agents may be needed in such cases until the pressure rebounds. Conversely, an “ultra-short-acting” CCB has a quick onset and quick offset: BP typically recovers in minutes (not hours) after discontinuation or down-titration. In ACCELERATE, 3 of 37 patients receiving clevidipine developed mild/moderate hypotension, all of which resolved with dose reduction or drug discontinuation. Rates of hypotension with nitroprusside are around 5% and, with nicardipine, up to 7.9%, according to a systematic review of antihypertensives in acute neurovascular emergencies.

Although time-to-onset and safety of antihypertensives are critical in both ICH and acute ischemic stroke (AIS), there are other variables to consider. For example, maintaining a steady SBP during a continuous infusion may affect functional outcomes, quality of life, or long-term disability. Quality improvement projects may show that minimizing the need for dynamic BPV control, frequent dose adjustments, and use of rescue therapies may reduce strain on hospital resources, volume overload events, intubations, length of stay, and/or financial burden… or may not… we do not know yet. Hence, on the topic of BPV in stroke, a panoply of research avenues abounds!

The very first BPV researcher may have been English anatomist Stephen Hales (1677–1761), who authored a treatise on the rise and fall of BP when he visually inspected and measured levels of pulsating blood in a glass tube inserted into a horse’s femoral artery [12]. As blood levels fluctuated several inches up and down, Hales recorded this nuanced observation in 1733 and therefore may be the original genius to describe BPV, calling it “Haemastaticks” [12]. Since then, BPV has been shown to occur not only physiologically as a diurnal phenomenon when we awaken and go to sleep but also as a pathological entity, having been reported in various disease processes as well. With Hales in mind, our 2025 consensus statement [1] nearly 300 years after his initial observations, was intended to outline a future (2030?) research framework and agenda, not to function as a prescriptive guideline or “recommendation” that should be applied in clinical practice now. We wrote this plainly and unambiguously: prospective studies are essential to refine definitions, validate metrics and thresholds, evaluate pharmacologic strategies, and corroborate the clinical relevance of BPV. Upcoming research worth mentioning is the Clevidipine for the Antihypertensive Treatment of Acute Intracerebral Hemorrhage (CLUTCH) trial, which aims to “compare the rate of hypertensive subjects with ICH who reach SBP target with stability within 60 min of enrollment, among patients treated with IV clevidipine with those treated with alternate IV antihypertensive regimen” [13]. Another area of stroke-hypertension research is prehospital: for guideline-directed SBP lowering prior to hospital arrival, look to a “Goldilocks approach: not too high and not too low”—if feasible, use of an ultra-short-acting CCB inside the ambulance may trim time in the emergency department spent lowering BP to reach AHA/ASA goals for thrombolytic in AIS [14]. Conceivably, mitigating peri-thrombolytic BPV may offer benefit as well, but again, this is just a hypothesis at this point [14].

In closing, we can all agree that for ICH and AIS, multiple studies have already reported that higher BPV is linked to worse outcomes, and so, therefore, it is worth investigating whether this is merely an association or, instead, a negative risk factor that, when modified, can improve lives. As our understanding of BPV evolves, we predict that future data will add to the concepts that we have proposed in our *Viewpoint*. It is exciting that there is interest from readers in this framework for research, and we value ongoing dialogue.

## Source of support

None.
